# Intraoperative implantation of 125I seeds improves prognosis in refractory stage IIIB cervical cancer: a case report and literature review

**DOI:** 10.1186/s12905-024-02997-1

**Published:** 2024-03-02

**Authors:** Jialin Wu, Junying Tang, Yi Luo, Wenbo Li, Yingwei Liu, Lin Xiao

**Affiliations:** 1 Department of Gynecology, the First Affiliated Hospital of Chongqing Medical University, Chongqing, 400016 China; 2 Department of Oncology, the First Affiliated Hospital of Chongqing Medical University, Chongqing, 400016 China; 3 Department of Nuclear Medicine, the First Affiliated Hospital of Chongqing Medical University, Chongqing, 400016 China

**Keywords:** Advanced cervical cancer, Radiotherapy, 125I seeds implantation, Radical hysterectomy, Prognosis

## Abstract

**Background:**

Concurrent chemoradiation is the standard treatment for advanced cervical cancer. However some patients still have a poor prognosis, and currently, there is no effective treatment for recurrence. In recent years, 125I seed implantation therapy has emerged as a treatment for advanced malignant tumors including surgically unresectable tumors, residual tumors after surgical resection, and metastatic tumors. However, the use of 125I seeds implantation in primary advanced cervical cancer has not been reported. In this study, we present a case of stage IIIB cervical cancer in a patient who had poor response to radiotherapy and chemotherapy. Subsequently, a radical hysterectomy was performed, and 125I radioactive seeds were successfully implanted during the surgery. This effectively controlled the lesions that were resistant to radiotherapy and had the potential to improve the prognosis.

**Case presentation:**

A 56-year-old woman was diagnosed with stage IIIB (FIGO 2009) IIIC1r (FIGO 2018) squamous carcinoma of the cervix. After receiving 4 cycles of platinum-based chemotherapy and 30 rounds of radiotherapy, she underwent a radical hysterectomy. The localized cervical lesions were reduced, but there was no reduction in the size of the enlarged pelvic lymph nodes. Therefore, 125I seed implantation was performed under direct surgical vision for the right paracervical lesion and the enlarged pelvic lymph nodes on the right side. During the 18-month follow-up period, the enlarged lymph nodes subsided without any signs of recurrence or metastasis.

**Conclusion:**

Intraoperative implantation of 125I seeds in lesions that are difficult to control with radiotherapy or in sites at high risk of recurrence is a feasible and effective treatment option for patients with advanced squamous cervical cancer, and it may contribute to improved survival.

## Background

Cervical cancer ranks as the fourth most common cancer and the fourth leading cause of cancer-related deaths in women, with an estimated 604,000 new cases and 342,000 deaths worldwide in 2020 [1]. The primary objective in the management of all gynecological cancers is to identify and treat precancerous lesions, intending to enhance patient prognosis through prevention and early detection [2]. Surgical intervention serves as the primary approach for treating early-stage cervical cancer, and the use of minimally invasive surgery does not have an adverse impact on survival outcomes in low-risk populations [3]. Concurrent chemoradiation is considered the standard of care for advanced cervical cancer, specifically stages IIB-IV.

Advanced cervical cancer has a bleak outlook because of paracervical invasion, lymph node metastasis, or distant metastasis, with 5-year survival rates of 55%, 35%, and 15% for FIGO (International Federation of Gynecology and Obstetrics) stages II, III, and IVA, respectively [4]. The estimated recurrence rate for FIGO stage III is about 42% [5]. Treatment options for recurrent or metastatic cervical cancer are limited and pose a difficult and complex challenge. One safe and successful treatment option for recurrent vulvar cancer is electrochemical therapy. It can also be used as a part of non-standard treatment for recurrent cervical cancer [6].

125I seeds belong to low-dose-rate brachytherapy which offers advantages such as high precision, strong adaptability, and minimal peripheral organ damage. It can effectively inhibit the proliferation of tumor cells and promote apoptosis [7]. This treatment method is widely used for various tumors, including brain tumors, eye tumors, lung cancer, hepatocellular carcinoma, prostate cancer, and more [8]. However, its successful application in cervical cancer is rare, and the current reports are limited to patients with recurrent cervical cancer.

We present a case report of a patient with stage IIIB squamous cervical cancer. Initially, the patient received radiotherapy but unfortunately experienced a poor outcome. To address this, a radical hysterectomy was then performed. During the surgery, successful intraoperative implantation of 125I radioactive seeds took place. The innovative approach effectively controlled the lesions that were previously unresponsive to conventional radiotherapy. Additionally, it reduced the risk of recurrence at high-risk sites and ultimately improved the patient’s prognosis.

## Case presentation

**Fig. 1:**
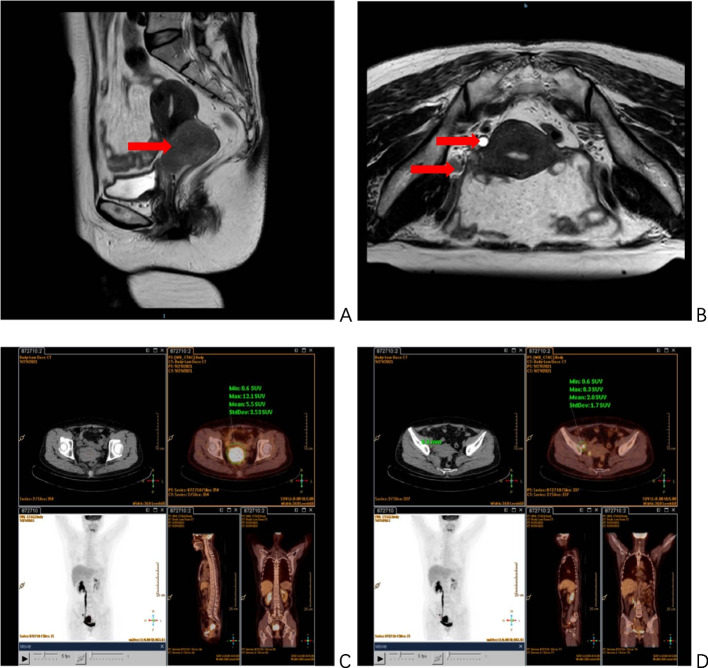
MRI before receiving treatment: **A** cervical occupation (arrowed), measuring about 4.5*4.6*4.8 cm, involving the external cervical os and poorly demarcated from the vagina; **B** an enlarged lymph node shadow is seen on the right side of the pelvic wall (arrowed); the right pelvic segment of the ureter is involved with fluid retention (arrowed). PET-CT before receiving treatment: **C** the cervix is significantly enlarged in size and metabolic activity, the lesion is poorly demarcated from the pelvic segment of the right ureter, with dilated ureter and renal pelvis hydronephrosis at its upper end; **D** metabolic activity of lymph nodes on the right side of the pelvic wall is increased, and metastasis is considered

The patient, a 56-year-old woman, was admitted to The First Affiliated Hospital of Chongqing Medical University on September 29, 2021. She presented with a history of vaginal discharge lasting more than two months and had received a recent diagnosis of cervical squamous carcinoma three days before admission. The patient also has underlying hypertension, but her blood pressure is under control. Examination Findings: The vulva showed normal development. The vagina appeared smooth, with a small amount of bright red blood present. The cervix exhibited a cauliflower-like neoplasm measuring approximately 5*5*4cm, which caused bleeding upon palpation. The uterus was positioned anteriorly and showed signs of atrophy. On the right side, there was thickening extending up to the pelvic wall, while no obvious abnormalities were observed on the left side. The patient was infected with HPV type 16. Squamous cell carcinoma antigen (SCC-Ag) was measured at 54.9ng/ml, which exceeds the normal range of 0–2.7ng/ml. Based on imaging and pathological examination, the patient was diagnosed with stage IIIB cervical squamous carcinoma (according to FIGO 2009 staging system). The diagnosis was supported by the presence of a lesion involving the right ureteropelvic segment, leading to dilated hydronephrosis of the right ureter and renal pelvis. Additionally, the patient was found to have stage IIIC1r disease (according to the FIGO 2018 staging system), characterized by enlarged and metabolically active right-sided pelvic closed-cell lymph nodes. These lymph nodes were determined to be metastatic on the PET-CT, as shown in Fig. 1. Three days after biopsy confirmation of diagnosis, the patient underwent a TP protocol consisting of four doses of intravenous chemotherapy, which included paclitaxel at a dosage of 240 mg and nedaplatin at a dosage of 130 mg. External radiation radiotherapy was given approximately 4 weeks after completion of chemotherapy. The prescribed dose for the metastatic lymph node gross tumor volume (GTVnd) was 64 Gy, delivered in 2.13 Gy fractions throughout 30 treatments. The prescribed dose for Clinical Target Volume (CTV) was 56 Gy, delivered in 2.0 Gy fractions throughout 28 treatments. The prescribed dose for Planning Target Volume (PTV) was 50.40 Gy, delivered in 1.8 Gy fractions throughout 28 treatments.

**Fig. 2:**
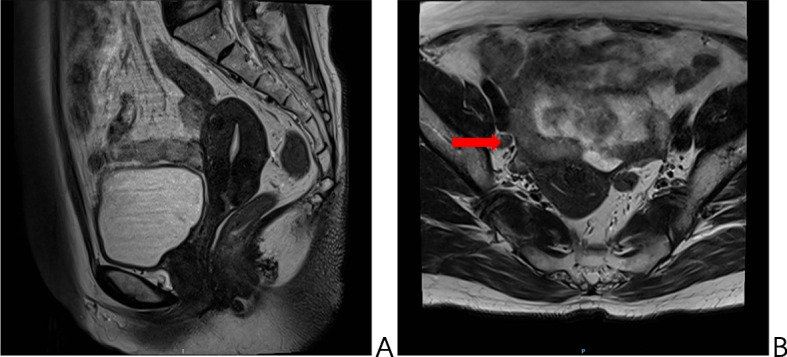
Post-radiotherapy (pre-surgery) MRI: **A** localised lesion significantly reduced. **B** enlarged lymph nodes on the right side (shown by arrows), insignificantly changed from before

**Fig. 3:**
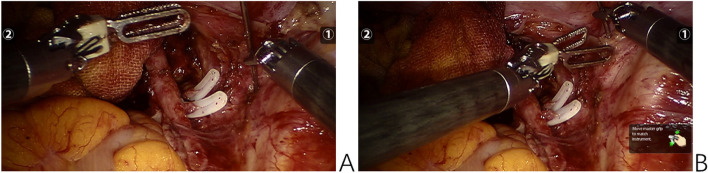
Intraoperative images of implanted 125I seeds

Upon assessment after the therapeutic intervention, it was observed that the local lesions had decreased in size (Fig. 2). Additionally, imaging results showed that the pelvic lymph nodes, which had enlarged before treatment, had not reduced in size despite radiation and chemotherapy. To achieve optimal lesion control and reduce the risk of recurrence, a comprehensive treatment plan was developed in collaboration with the patient’s surgical preferences. Consequently, a radical hysterectomy with bilateral adnexectomy and 125I seed implantation was performed on March 9, 2022 (approximately 5 weeks after completion of radiotherapy), using the da Vinci robot-assisted laparoscopic technique. This procedure involved both gynecology and nuclear medicine. During the surgery, it was noted that the uterus was centrally positioned and normal in size. However, it was fixed on the right side and accompanied by a thickening of the right parietal tissue. There was also a significant presence of hydrosalpinx, through which the right ureter passed. Additionally, the right external iliac vessels and internal iliac vessels were closely situated and could not be separated. The procedure initially involved a radical hysterectomy. During the surgery, a total of 30 125I seeds were implanted in the para-uterine and right occluded lymph node region (Fig. 3), targeting areas where tumors may be uncontrolled and recurrent, as confirmed by imaging. The decision to implant the seeds was based on several factors, including dense adhesions in the right pelvic wall, the relationship between the right para-uterine lesion and the ureteral bladder, the lack of significant shrinkage in the swollen pelvic lymph nodes after preoperative radiotherapy, and the platelike nature of the right pelvic wall, which made it difficult to separate the right external iliac and internal iliac vessels. 18 G implantation needles were used to directly insert the seeds into the target lesions, avoiding large blood vessels and nearby ureters. A turntable gun was then utilized to place the 125I seeds into the para-uterine and right occluded lymph node region, with seeds spaced 0.5–1 cm apart upon withdrawal of the needles. Pelvic lymphadenectomy was not performed due to a frozen pelvis resulting from radical radiotherapy, which made dissection of the iliac vessels and obturator nerves challenging. The surgical procedure was completed with an intraoperative hemorrhage volume of 50 ml. The patient had a favorable recovery with no postoperative complications and was discharged from the hospital on the fifth day after the procedure. Pathological examination results confirmed that the cervix was thoroughly examined and showed no evidence of residual cancer. Focal tissue calcification and a post-chemotherapy reaction were identified. No cancer involvement was observed in the left and right parietal uterus, sacral ligament, and incision margin of the vaginal wall.

**Fig. 4:**
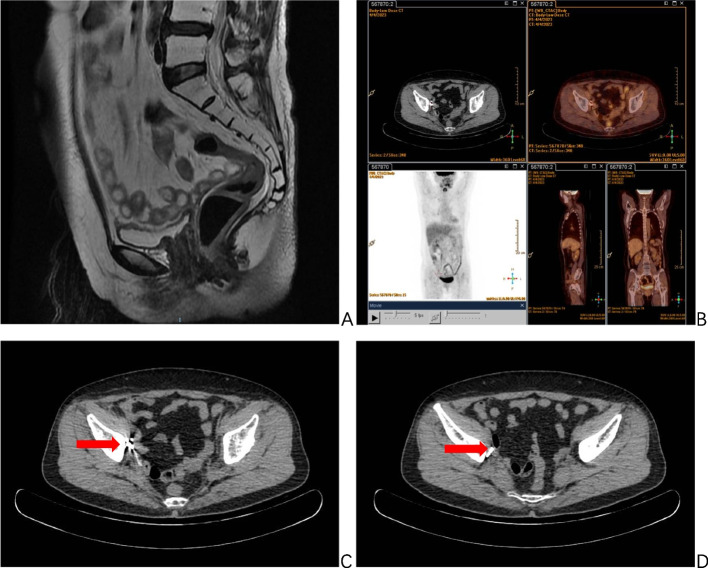
MRI and PET-CT examination after 125I seeds implantation. MRI examination about 6 months after 125I seeds implantation is shown in Figure **A** T2W sagittal position, the uterus, and bilateral adnexa were not shown, the uterus was altered postoperatively, and there were no definite signs of tumor recurrence in the operated area. PET-CT examination 13 months after 125I seeds implantation is shown in Figure **B** multiple punctate dense shadows on the right wall of the pelvis, and no swollen lymph nodes were seen in the pelvis and bilateral inguinal area, and bilateral inguinal area showed no enlarged lymph nodes. Figures **C** and **D** multiple punctate dense shadows in the right wall of the pelvis (indicated by arrows), which are seeds implantation changes

Following the surgical procedure, the patient underwent two cycles of intravenous chemotherapy using the TP protocol. This protocol consisted of administering paclitaxel at a dosage of 240 mg and nedaplatin at a dosage of 130 mg. Regular follow-up examinations were conducted every 3 months, which included cytology, colposcopy, tumor indicators (SCC), and imaging techniques such as MRI and PET-CT (Fig. 4). The objective of these examinations was to monitor the local control of the lesion and detect any presence of enlarged lymph nodes. It has been 18 months since the implantation of 125I seeds, and there is no evidence of tumor recurrence or metastasis.

## Discussion

Concurrent chemoradiation plays a crucial role in the treatment of advanced cervical cancer. Recent years have seen several randomized clinical trials that demonstrate the efficacy of concurrent chemoradiation in improving both overall survival (OS) and progression-free survival (PFS) in locally advanced cervical cancer (stage IIB III IVA) [9–11]. There are still some patients, however, whose lesions are difficult to control or recur, leading to a poor prognosis. Treatment options for recurrent cervical cancer are limited and challenging. The main approaches include systemic chemotherapy, radiotherapy, surgical treatment (pelvic exenteration), and targeted or immunotherapies. Systemic chemotherapy, while palliative, cannot achieve a cure and may be limited by adverse side effects or drug resistance. A new strategy, targeted or immunotherapy, is currently in clinical trials for most medications [12]. The effectiveness of treatment remains uncertain. Pelvic exenteration is a complex and demanding intervention, with a perioperative mortality rate ranging from 2 to 5% and a surgical complication rate ranging from 32 to 84% [13]. These complications mainly include wound-healing disorders, ileus complaints, intra-abdominal abscesses or anastomotic insufficiencies, fistulas, and ureteral stenosis, resulting in prolonged hospital stays and poor quality of life after surgery. Furthermore, some patients still face the risk of recurrence [14]. Vivod G et al. showed that electrochemotherapy might be a less radical alternative to pelvic exenteration, which is feasible and suitable for the treatment of recurrent vulvar cancer. Electrochemical therapy may be used for the treatment of locally recurrent cervical cancer [15].

In recent years, 125I seed implantation therapy has emerged as a treatment for advanced malignant tumors, which include surgically unresectable tumors, residuals after surgical tumor resection, and metastatic tumors. This technique is now extensively utilized in the treatment of various types of tumors, such as brain tumors, eye tumors, lung cancer, hepatocellular carcinoma, prostate cancer, and more [8]. With high precision, adaptability, and minimal damage to peripheral organs, this treatment method can effectively reduce tumor burden and improve clinical symptoms, particularly pain. This treatment has shown significant advantages with a relief rate of 81.3% [7]. Other symptoms such as fatigue (75.6%), bowel abnormality (33.3%), abdominal distension (30.3%), and urinary irritation (36.3%) also demonstrated varying degrees of relief, greatly enhancing the patient’s quality of life [7].

125I seed implantation is typically performed under CT or ultrasound guidance, and intraoperative implantation is less commonly reported. In our previous study, we reported a case of a patient with recurrent epithelial ovarian cancer who showed an improved prognosis after early diagnosis of recurrence and a second tumor cytoreduction in 2018. During this procedure, 125I seeds were implanted in a difficult-to-resect lesion located around the right ureter and right external iliac vessel. The patient has survived until the present day [16]. In recent years, it has been demonstrated that 125I seed implantation can be effectively treat recurrent cervical cancer, leading to improved survival and long-term outcomes for patients [7, 17]. Wei and others [18] also reported a case of immunotherapy combined with 125I seed implantation for the treatment of recurrent cervical cancer following concurrent radiotherapy. This study showed that 125I seed implantation was a safe and effective therapeutic strategy, suggesting its promising potential. Dealing with recurrent cervical cancer is challenging, and the primary goal of treatment is to prolong patient survival. Some studies have proposed that 125I particle implantation could be a reliable comprehensive treatment for primary cervical cancer [19]. However, there have been no reports of 125I seed implantation being applied to primary advanced cervical cancer.

Additionally, there is a limited body of research on the topic of adjuvant surgery following radiotherapy. A study on concurrent radiotherapy combined with surgery for stage IIB-IIIB cervical cancer revealed that this treatment approach resulted in a decrease in the rate of postoperative recurrence. Additionally, the study found no significant rise in postoperative complications [20].

The advantages of our case include direct surgical visualization during the implantation of 125I seeds. The efficacy of 125I brachytherapy depends on the size of the tumor size and the precise placement of the radioactive particles [21]. In this particular instance, the 125I radioactive particles were implanted with direct surgical visualization, ensuring a higher level of accuracy and safety. Secondly, the utilization of radiotherapy in conjunction with adjuvant surgery. After completing radiotherapy, a Radical Hysterectomy was performed on the patient in light of imaging findings indicating uncontrolled lesions (pelvic metastatic lymph nodes). Additionally, seeds were implanted in the para-uterine area at risk of recurrence, resulting in a reduction in the recurrence rate [20]. The study has certain limitations, namely its retrospective nature and reliance on case reports from a single institution. Additionally, the duration of patient follow-up was relatively short. Therefore, it is imperative to conduct additional research involving a larger sample size of patients and implement long-term monitoring of this treatment to establish conclusive findings. This procedure deviates from the standard protocol as we chose not to conduct pelvic lymphadenectomy due to the presence of a frozen pelvis resulting from radical radiotherapy. This condition posed challenges in dissecting the iliac vessels and obturator nerves. Radiotherapy, when combined with adjuvant surgery involving intraoperative implantation of 125I seeds, has the potential to improve the prognosis of patients with stage IIIB cervical cancer.

## Conclusion

Radiotherapy, in combination with radical hysterectomy and intraoperative implantation of 125I seeds, can be applied to the treatment of patients with advanced squamous carcinoma of the cervix, it is safe, effective, and has the potential to improve prognosis. The presented case report serves as a valuable reference for the management of advanced squamous carcinoma of the cervix. Further investigations involving a more extensive patient cohort and extended follow-up are necessary to establish definitive conclusions regarding the efficacy of this treatment.

## Data Availability

Data is contained within the article.
